# Rapid, Specific Detection of Alphaviruses from Tissue Cultures Using a Replicon-Defective Reporter Gene Assay

**DOI:** 10.1371/journal.pone.0033007

**Published:** 2012-03-12

**Authors:** Jiangjiao Li, Wuyang Zhu, Huanqin Wang, Jiandong Li, Quanfu Zhang, Ying He, Jia Li, Juanjuan Fu, Dexin Li, Guodong Liang

**Affiliations:** 1 Department of Viral Encephalitis, Institute for Viral Disease Control and Prevention, Chinese Center for Disease Control and Prevention (IVDC, China CDC), Beijing, China; 2 State Key Laboratory for Infectious Disease Prevention and Control (SKLID), Department of Viral Hemorrhagic Fever (IVDC, China CDC), Beijing, China; Naval Research Laboratory, United States of America

## Abstract

We established a rapid, specific technique for detecting alphaviruses using a replicon-defective reporter gene assay derived from the Sindbis virus XJ-160. The pVaXJ expression vector containing the XJ-160 genome was engineered to form the expression vectors pVaXJ-EGFP expressing enhanced green fluorescence protein (EGFP) or pVaXJ-GLuc expressing *Gaussia* luciferase (GLuc). The replicon-defective reporter plasmids pVaXJ-EGFPΔnsp4 and pVaXJ-GLucΔnsp4 were constructed by deleting 1139 bp in the *non-structural protein 4* (*nsP4*) gene. The deletion in the *nsP4* gene prevented the defective replicons from replicating and expressing reporter genes in transfected BHK-21 cells. However, when these transfected cells were infected with an alphavirus, the non-structural proteins expressed by the alphavirus could act on the defective replicons in *trans* and induce the expression of the reporter genes. The replicon-defective plasmids were used to visualize the presence of alphavirus qualitatively or detect it quantitatively. Specificity tests showed that this assay could detect a variety of alphaviruses from tissue cultures, while other RNA viruses, such as Japanese encephalitis virus and Tahyna virus, gave negative results with this system. Sensitivity tests showed that the limit of detection (LOD) of this replicon-defective assay is between 1 and 10 PFU for Sindbis viruses. These results indicate that, with the help of the replicon-defective alphavirus detection technique, we can specifically, sensitively, and rapidly detect alphaviruses in tissue cultures. The detection technique constructed here may be well suited for use in clinical examination and epidemiological surveillance, as well as for rapid screening of potential viral biological warfare agents.

## Introduction

Alphaviruses are a group of 30 mosquito-borne arboviruses belonging to the genus *Alphavirus* in the family *Togaviridae* [1]–[3]. Some alphaviruses, such as Sindbis virus (SINV) [4] and Chikungunya virus (CHIKV) [5], [6], can cause fever, arthritis, rash, and other symptoms. Alphaviruses such as Western equine encephalitis virus (WEEV) [7], Eastern equine encephalitis virus (EEEV) [8], and Venezuelan equine encephalitis virus (VEEV) [9] can cause fever and viral encephalitis in humans. Another alphavirus, Getah virus (GETAV), causes pyrexia, skin eruptions, edema of the hind limbs, and enlargement of the submandibular lymph nodes in horses or swine [10]. Alphaviruses are distributed globally, and have caused several epidemics, including the large-scale CHIKV infection in the Indian Ocean region in 2005 and the ensuing global infection [11]–[13]. The high pathogenicity of alphaviruses makes them a substantial international public health threat; consequently, a variety of alphaviruses (EEEV, WEEV, and VEEV) have been classified as potential biological warfare agents that could threaten public security [14]. Therefore, a rapid, specific detection technique for alphavirus pathogens plays an important role in monitoring infectious diseases and public security.

Recently, several virus detection techniques have been developed using virus-specific reporter gene assays that efficiently and sensitively detect the corresponding viruses, such as rubella and herpes simplex [15]–[20]. In this study, we established a rapid, specific method for detecting alphaviruses using a reporter gene assay that uses a defective replicon derived from the XJ-160 virus. In this assay, the structural genes of SINV were replaced by reporter genes, and a deletion was made in the *non-structural protein 4* (*nsP4*) gene to construct defective replicons of SINV. On infection with an alphavirus, the alphavirus components induce the expression of the reporter gene in the cells transfected with the replicon-defective plasmid. This simple, rapid, specific method using defective replicons can quickly discriminate whether an unknown sample contains an alphavirus from the first passage in tissue cultures.

## Results

### Characteristics of reporter gene expression by XJ-160 replicons

**Figure 1:**
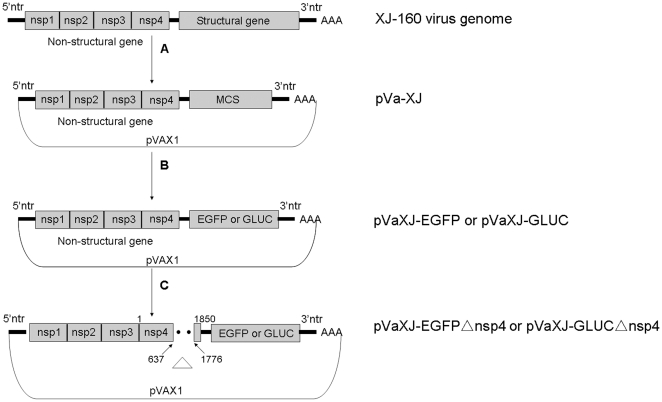
Diagram of the generation of defective XJ-160 replicons. (A) The pVa-XJ replicon was constructed by inserting the XJ-160 virus genome into the eukaryotic expression vector pVAX1 and replacing the structural gene with the multiple cloning site (MCS) sequence. (B) The constructs containing the reporter gene (pVaXJ-EGFP or pVaXJ-GLuc) were generated by digesting the *enhanced green fluorescent protein* (*EGFP*) gene or *Gaussia luciferase* (*GLuc*) gene with the restriction enzymes *Fse*I and *Asc*I, and ligating them into the MCS of pVa-XJ. (C) The defective XJ-160 replicons (pVaXJ-EGFPΔnsp4 and pVaXJ-GLucΔnsp4) were produced by introducing an 1139-nt deletion mutation in the non-structural protein coding regions of pVaXJ-EGFP and pVaXJ-GLuc using *Acl*I digestion. The deleted regions of the XJ-160 genome in defective replicons are denoted by dotted lines and the non-structural protein gene deletion is designated by a “Δ”.

To generate defective replicons used for detecting alphaviruses, we first constructed the plasmids pVaXJ-EGFP and pVaXJ-GLuc containing the XJ-160 virus, a SINV isolated from Xinjiang, China [21], and reporter genes that express enhanced green fluorescence protein (EGFP) or *Gaussia* luciferase (GLuc), respectively. Then, the replicon-defective plasmids pVaXJ-EGFPΔnsp4 and pVaXJ-GLucΔnsp4 were constructed by digesting pVaXJ-EGFP or pVaXJ-GLuc with *Acl*I to delete 1139 bp from the *nsP4* gene (Figure 1).

**Figure 2:**
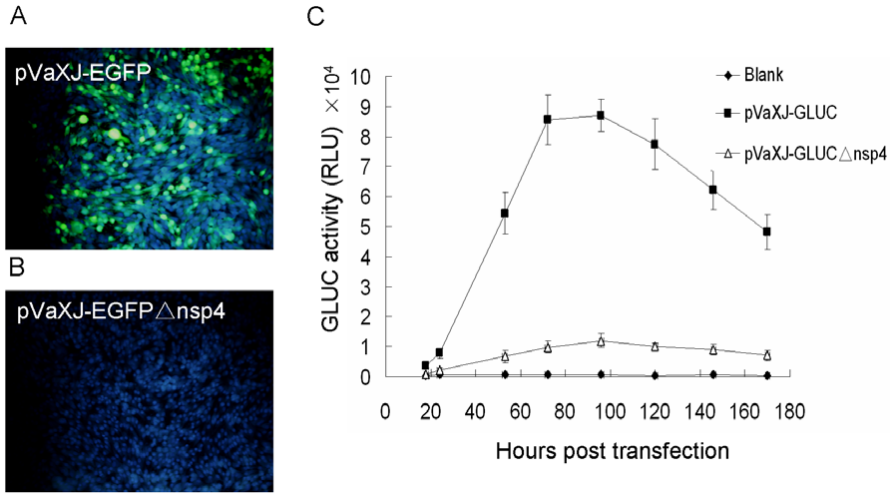
EGFP and GLuc expression in replicon-transfected cells. BHK-21 cells were transfected with pVaXJ-EGFP, pVaXJ-EGFPΔnsp4, pVaXJ-GLuc, or pVaXJ-GLucΔnsp4 using Lipofectamine 2000 reagent. (A), (B) EGFP expression was examined 48 h post-transfection using an Olympus IX51 fluorescence microscope. Green color indicates EGFP, and blue color indicates nucleus. (C) GLuc activity was measured at different time points (from 18–170 h) post-transfection using the BioLux™ Gaussia Luciferase Assay Kit and a luminometer. Each data point represents the mean ±SEM of three independent experiments. RLU, relative light units.

Reporter gene expression assays showed that specific green fluorescence was observed after BHK-21 cells were transfected with pVaXJ-EGFP (Figure 2A), while no EGFP expression was observed following transfection with the defective replicon pVaXJ-EGFPΔnsp4 (Figure 2B). Similarly, high luciferase activity was detected after BHK-21 cells were transfected with the pVaXJ-GLuc replicon, while only background levels of luciferase were detected after transfection with the defective replicon pVaXJ-GLucΔnsp4 (Figure 2C). These results indicate that the defective replicons did not express reporter genes normally because of the 1139 bp deletion in the nsP4 region.

### Detection capability of defective replicon cassettes

**Figure 3:**
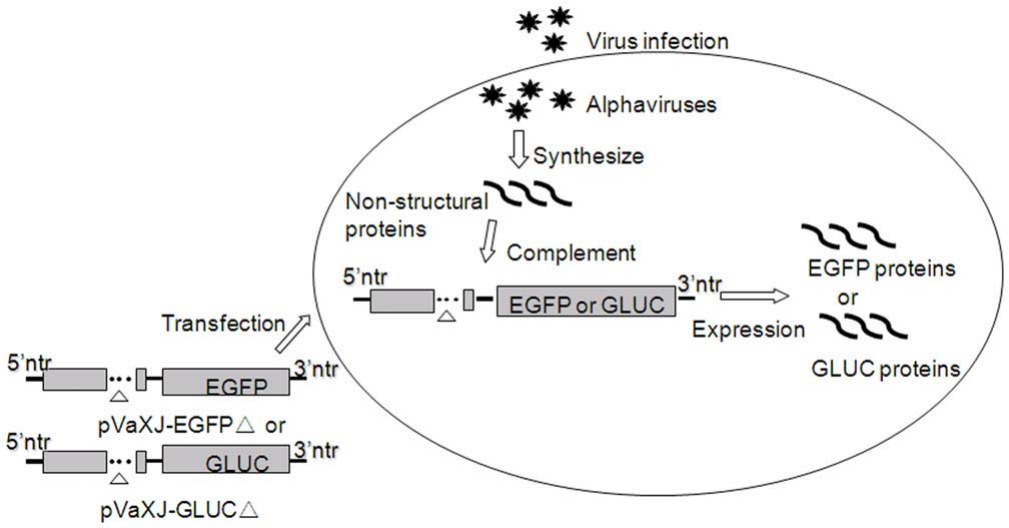
Schematic diagram of *Alphavirus* detection using defective XJ-160 replicons. Due to the deletion in the non-structural protein coding region, the defective XJ-160 replicons (pVaXJ-EGFPΔ nsp4 and pVaXJ-GLucΔ nsp4) failed to express the reporter genes efficiently. When cells were infected with alphavirus, the alphavirus synthesized the viral non-structural proteins, and these proteins acted on the defective XJ-160 replicons in *trans*, resulting in high-level expression of the reporter genes, which could be easily measured.

As mentioned previously, because of the deletion in the *nsP4* gene, the defective replicons could not replicate and express reporter genes normally after being introduced into cells. However, when transfected cells were infected with an alphavirus, the non-structural protein expressed by the infecting alphavirus could act on the defective replicons in *trans* and result in the expression of the reporter genes. Using this reporter assay, an alphavirus in an undefined sample should be detected by observing the expression of the reporter genes. A schematic diagram of alphavirus detection using defective replicons is shown in Figure 3.

**Figure 4:**
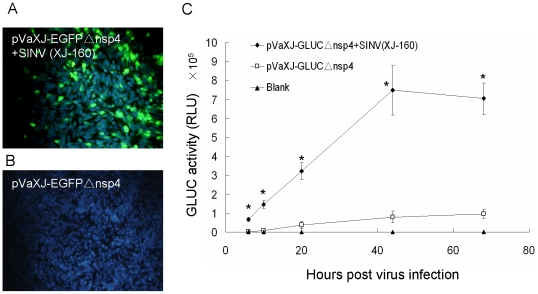
EGFP and GLuc expression in replicon-defective transfected cells infected with SINV (XJ-160). BHK-21 cells were transfected with pVaXJ-EGFPΔnsp4 or pVaXJ-GLucΔnsp4 using Lipofectamine 2000 reagent, 6 h before mock-infection or infection with 1 MOI SINV (XJ-160). (A, B) EGFP expression was examined 44 h post-infection. Green color indicates EGFP, and blue color indicates nucleus. (C) GLuc activity was measured at different time points (from 6–68 h) post-infection. *p<0.05, (pVaXJ-GLucΔnsp4+SINXJ160) vs. pVaXJ-GLucΔnsp4.

To confirm the detection capability of the defective replicons, BHK-21 cells were transfected with pVaXJ-EGFPΔnsp4 and then infected with 1 MOI of XJ-160 virus. EGFP expression could be observed 44 h post-infection under a fluorescence microscope (Figure 4A), while no expression of EGFP was observed in the control group with no virus infection (Figure 4B). Luciferase assays indicated that the luciferase activity could be detected 6 h post-infection, reaching a peak value at 44 h post-infection, and gradually decreasing after 44 h. The luciferase activity 44 h post-infection in the experimental group was 10 times higher than that in the pVaXJ-GLucΔnsp4 control group (Figure 4C). Both the qualitative and quantitative results suggest that the defective replicon-defective reporter gene assay could detect SINV XJ-160 in infected cells.

### Specificity of the replicon-defective alphavirus detection method

**Figure 5:**
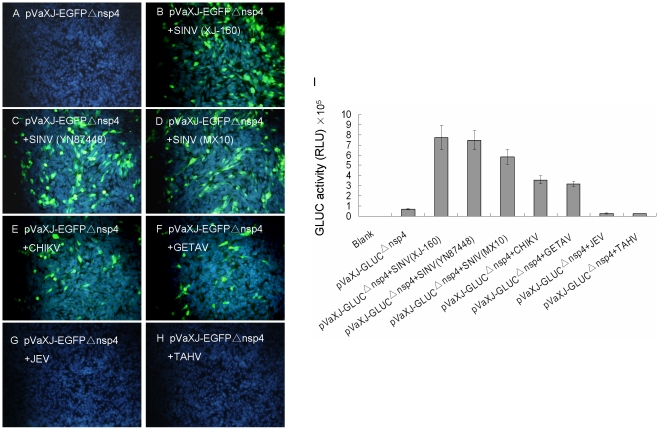
EGFP and GLuc expression in replicon-defective transfected cells infected with various viruses. BHK-21 cells were transfected with pVaXJ-EGFPΔnsp4 or pVaXJ-GLucΔnsp4 using Lipofectamine 2000 reagent, 6 h before mock-infection or infection with 1 MOI of various viruses, including alphaviruses [SINV (XJ-160, YN87448, and MX10), CHIKV (SD08Pan), and GETAV (HB0234)], and non-alphaviruses [JEV (P3) and TAHV (XJ0625)]. (A–H) EGFP expression was examined 44 h post-infection. Green color indicates EGFP, and blue color indicates nucleus. (I) GLuc activity was measured 44 h post-infection. Each data point represents the mean ±SEM of three independent experiments. RLU, relative light units.

To determine the specificity of the replicon-defective alphavirus detection method, we attempted to detect a variety of alphaviruses and non-alphaviruses. EGFP expression assays indicated that BHK-21 cells produced green fluorescence when the reporter-transfected cells were infected with three strains of SINV (XJ-160, YN87448, and MX10), CHIKV (SD08Pan), and GETAV (HB0234) (Figure 5B, 5C, 5D, 5E, 5F), and that no green fluorescence was observed after infection with the Japanese encephalitis virus (JEV, P3) or Tahyna virus (TAHV, XJ0625) (Figure 5G, 5H). The luciferase activity assay showed that GLuc expression activity was significantly increased in the alphavirus infection group, compared to pVaXJ-GLucΔnsp4 in the control group without any virus infection. The reporter plasmid-transfected BHK-21 cells infected with XJ-160, YN87448, MX10, SD08Pan, or HB0234 virus expressed GLuc with activities that were 10.2, 9.1, 7.4, 5.6, and 5.3 times higher, respectively, than that of the control group. Following infection with JEV (P3) or TAHV (XJ0625) viruses, the GLuc activities were similar to that of pVaXJ-GLucΔnsp4 in the control group (Figure 5I). These results show that the defective replicon-based method can detect alphaviruses from tissue culture, while other RNA viruses, such as the P3 flavivirus and XJ0625 bunyavirus gave negative results with this system.

### Sensitivity of the replicon-defective alphavirus detection method

**Figure 6:**
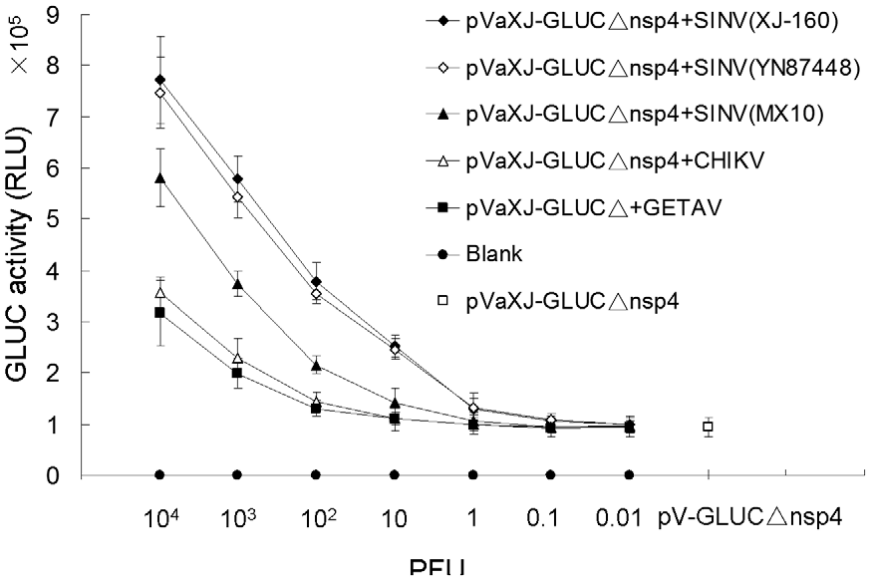
GLuc expression in replicon-defective transfected cells infected with varying PFUs of alphaviruses. BHK-21 cells were transfected with pVaXJ-GLucΔnsp4 using Lipofectamine 2000 reagent, 6 h before mock-infection or infection with varying PFU levels of alphaviruses. GLuc activity was measured 44 h post-infection. Each data point represents the mean ±SEM of three independent experiments. RLU, relative light units.

To determine the sensitivity of the alphavirus detection method, several alphaviruses with a titer of 1×10^5^ PFU/ml were serially diluted (from 10^−1^ to 10^−7^), resulting in the addition of 10^4^, 10^3^, 10^2^, 10, 1, 0.1, and 0.01 PFU to the plates, respectively. BHK-21 cells, transfected with each of the defective replicons, were infected with the alphaviruses and assayed for expression of the reporter protein. Luciferase activity assays indicated that the GLuc activity reached a peak 44 h post-infection, and decreased gradually with the number of PFUs used for the infection. When the virus load was reduced to 1 PFU (XJ-160 or YN87448) or 10 PFU (MX10), the activity measured at 44 h was obviously above that of pVaXJ-GLucΔnsp4 in the control group (Figure 6), while the GLuc activity was enhanced until other alphaviruses increased to 10^2^ or 10^3^ PFU, indicating that the limit of detection (LOD) of this replicon-defective assay is between 1 and 10 PFU for Sindbis viruses and between 10^2^ and 10^3^ PFU for the two other alphaviruses.

## Discussion

All viruses have specific pathways for replication and expression of the viral RNA genome. Using this knowledge, it is possible to induce the specific expression of a reporter gene inserted into the viral genome. Therefore, under the control of virus-specific events, we can detect these viruses by measuring the reporter enzyme. This strategy provides a simple, effective, and virus-specific detection technique [22]–[24].

The genome structures and replication strategy specific to alphaviruses were used to construct an alphavirus-inducible reporter gene expression system. The alphavirus genome is a single strand of positive-sense RNA, which has two open reading frames: the 5′-end open reading frame that encodes non-structural proteins (nsP1–4) and the 3′-end open reading frame that encodes structural proteins (capsid proteins and envelope proteins). Non-structural proteins are the viral replicases/transcriptases, which are responsible for the replication and transcription of the viral genomic RNA, as well as the synthesis of subgenomic RNA [25]. nsP4 has RNA-dependent RNA polymerase activity, which is highly conserved in the genus *Alphavirus*, and plays a major role in viral RNA replication [26]–[28]. After an alphavirus enters a host cell, ribosomes bind to the positive-strand genomic RNA and produce non-structural proteins. Using the viral replicases, the negative-strand RNA is synthesized using the positive-strand RNA as a template. Next, subgenomic RNA is synthesized using the negative-strand RNA as a template. Finally, the subgenomic RNA is used as a template to translate and synthesize structural proteins to assemble virus particles [29]. The well-described genome structure and replication strategy of alphaviruses enabled us to construct a replication reporter system by replacing the structural genes with reporter genes to form the plasmids pVaXJ-EGFP and pVaXJ-GLuc. To form the replicon-defective reporter system, we deleted 1139 bp of the nsP4 coding frame using the *Acl*I site, forming the replicon-defective reporter plasmids pVaXJ-EGFPΔnsp4 and pVaXJ-GLucΔnsp4. As nsP4 plays an indispensable role in viral genome replication, the defective replicon could not replicate normally and express reporter genes after they were introduced into cells. By contrast, if the transfected cells were infected with an alphavirus, the nsP4 proteins produced by the alphavirus could act on the defective replicons in *trans*, leading to the expression of the reporter genes. In addition, the *nsP4* gene is highly conserved in the genus *Alphavirus* [30]. Recently, the nsP4 coding region of *Alphavirus* RNA was chosen as the target for genus-specific RT-PCR, and all alphaviruses, including Barmah Forest, Chikungunya, Mayaro, O'nyong-nyong, Ross River, Semliki Forest, Sindbis, Eastern equine encephalitis, Western equine encephalitis, and Venezuelan equine encephalitis viruses, were positive using the generic *Alphavirus* PCR [31]. Therefore, the defective replicons constructed using SINV XJ-160 can be used to detect a variety of alphaviruses.

We used the method to detect a variety of alphaviruses and non-alphaviruses from tissue culture. The results showed that the system could detect alphaviruses, including the Sindbis, Chikungunya, and Getah viruses. By contrast, the system gave a negative response to other RNA viruses, such as the Japanese encephalitis and Tahyna viruses. The system could detect alphaviruses with a viral load as low as 1–10^3^ PFU, indicating that this detection technique has a high specificity and sensitivity for alphaviruses. Sindbis viruses can be divided into three genotypes, the Palearctic/Ethiopian (P/E), Oriental/Australian (O/A), and Western-Australian (W/A), and the three strains of Sindbis viruses used in this study, YN87448, XJ-160, and MX10, belong to the P/E, W/A, and O/A genotypes, respectively [32]. Therefore, these strains represent the different known genotypes of SINV. Olivo *et al.* constructed a similar SINV defective replicon system, in which all four non-structural proteins gene were deleted [33]. Unlike the *nsP4* gene, the other three *nsP* genes share low homology among alphaviruses, so the detection system constructed by Olivo *et al.* could only be used to detect Sindbis virus, rather than other alphaviruses. In this study, we constructed a replicon-defective alphavirus detection system that is suitable for detecting a variety of alphaviruses, since we deleted only the majority of the nsP4 region, which is highly conserved in alphaviruses.

Presently, two methods are applied for the detection and identification of unknown viruses, including the detection of viral antigens and viral genes. To identify viral antigens, virus isolates must be incubated in sensitive cells, and then different virus antibodies are used to determine the virus type. This detection process is time consuming and it is difficult to determine the standard of the detection method. The PCR-based viral gene detection method is sensitive, but the chemical treatment of samples in PCR detection leads to viral lysis, making it impossible to isolate and culture the virus, which is undesirable when the virus sample is scarce. Compared with these methods, only a very small amount of sample needs to be incubated with cultured cells in our replicon-defective alphavirus detection method. In addition, the cytopathic effect (CPE) caused by alphavirus is usually observed more than 40 h post-infection [10], [21], [34], while our detection method can detect alphaviruses 6 h post-infection with the GLuc assay. Therefore, the replicon-defective alphavirus detection method can quickly, sensitively and specifically detect alphaviruses without affecting the viral multiplication capacity, allowing the isolation and cultivation of scarce viruses for use in subsequent studies.

We constructed a simple, rapid, color-visible replicon-defective detection assay for the detection of alphaviruses. These characteristics make it possible to use this detection technique in clinical examinations, epidemiological surveillance, and the rapid screening of viral biological warfare agents. If we select a packaging cell line that can express the defective replicon stably, the detection process will be even simpler and more convenient. The establishment of this detection method also provides a reference for the development of new detection methods for other RNA viruses.

## Materials and Methods

### Virus strains and cell culture

Sindbis viruses [XJ-160 strain (GenBank No. AF103728), YN87448 strain (GenBank No. AF103734), and MX10 strain], Getah virus [HB0234 strain (GenBank No. EU015062)], Japanese Encephalitis virus [P3 strain (GenBank No. U47032)], and Tahyna virus [XJ0625 strain (GenBank No. EU622820)] were isolated in China and are stored in our laboratory. Chikungunya virus [SD08Pan strain (GenBank No. GU199351)] was kindly provided by Professor Li, Institute for Viral Disease Control, Chinese Center for Disease Control. Baby hamster kidney (BHK-21) cells [21], [34] were maintained at 37°C under 5% CO_2_ in Dulbecco's Modified Eagle's Medium (DMEM) supplemented with 10% fetal bovine serum (FBS) and 100 U/ml each of penicillin and streptomycin.

### Construction of defective XJ-160 replicons

The vector pVaXJ, a DNA-based expression vector, was constructed by placing the recombinant genome of XJ-160 virus under the control of the human cytomegalovirus (CMV) promoter of plasmid pVAX1, in which the viral structural genes were replaced by a polylinker cassette to allow the insertion of heterologous genes. The cassettes pVaXJ-EGFP and pVaXJ-GLuc, expressing enhanced green fluorescence protein (EGFP) or *Gaussia* luciferase (GLuc), respectively, were constructed by cloning the *EGFP* or *GLuc* gene into pVaXJ. Then, the defective replicon-based plasmids pVaXJ-EGFPΔnsp4 and pVaXJ-GLucΔnsp4 were constructed by digesting pVaXJ-EGFP or pVaXJ-GLuc, respectively, with *Acl*I to delete 1139 bp in the *nsP4* gene, which is highly conserved in *Alphavirus* and plays a major role in viral RNA replication through its RNA-dependent RNA polymerase (RDRP) activity [26]–[28].

### Plaque assay

The observations of plaque morphology and determination of virus titer were performed using a plaque assay, as described previously [34]. Briefly, BHK-21 cells were grown to confluence in a 6-well plate to which 0.5 ml of diluted virus sample was added. After a 1-h incubation at 37°C, the supernatant was removed, and 2 ml of culture cover medium (2×DMEM containing 2% FBS equivalently mixed with 2% UltraPure agarose) were added. Then, the plate was turned over and incubated at 37°C in a humidified atmosphere with 5% CO_2_. When the cytopathic effect was seen under a microscope, another 2 ml of culture cover medium containing 10% neutral red (Sigma, USA) was added and the plates were incubated at 37°C under 5% CO_2_ for 6 h. The size and number of plaques were then recorded and the number of plaques was used to calculate the virus titer.

### Transfection and reporter gene expression

In 24-well plates, 1×10^5^ BHK-21 cells were transfected with 0.8 µg pVaXJ-EGFPΔnsp4 or pVaXJ-GLucΔnsp4 using Lipofectamine 2000 reagent (Invitrogen, USA), and the BHK-21 cells were infected with viruses at 6 h post-transfection. To observe EGFP expression, the medium was removed from the transfected cells and replaced with enough phosphate-buffered saline to keep the cells wet. The nucleuses were stained with 4′, 6-diamidino-2-phenylindole (DAPI). Then, the cells were examined using an Olympus IX51 fluorescence microscope. Micrographs were taken using an Olympus DP70 camera. To perform the GLuc activity assay, 20 µL of culture supernatant were collected at different time points and reacted with BioLux GLuc substrate in BioLux GLuc assay buffer from the BioLux™ Gaussia Luciferase Assay Kit (New England Biolabs). GLuc activity was detected by a Modulus luminometer (Promega), and the raw data, expressed as relative light units (RLU), were recorded.
