# Correction: Rapid, Specific Detection of Alphaviruses from Tissue Cultures Using a Replicon-Defective Reporter Gene Assay

**DOI:** 10.1371/annotation/f60cbe79-115a-4209-95f7-40cacca6b28a

**Published:** 2012-04-09

**Authors:** Jiangjiao Li, Wuyang Zhu, Huanqin Wang, Jiandong Li, Quanfu Zhang, Ying He, Jia Li, Juanjuan Fu, Dexin Li, Guodong Liang

There was an error in both affiliations. The correct affiliations are:

1 Department of Viral Encephalitis, Institute for Viral Disease Control and Prevention, Chinese Center for Disease Control and Prevention (IVDC, China CDC) and State Key Laboratory for Infectious Disease Prevention and Control (SKLID), Beijing, China,

2 Department of Viral Hemorrhagic Fever, IVDC, China CDC, Beijing, China 

